# From Identity to Uniqueness: The Emergence of Increasingly Higher Levels of Hierarchy in the Process of the Matter Evolution †

**DOI:** 10.3390/e20070533

**Published:** 2018-07-17

**Authors:** George Mikhailovsky

**Affiliations:** Global Mind Share, Norfolk, VA 23503-1312, USA; George@globalmindshare.org; Tel.: +1-(703)-801-6453

**Keywords:** identity, uniqueness, complexity, diversification, distinguishing, attraction, repulsion

## Abstract

This article focuses on several factors of complification, which worked during the evolution of our Universe. During the early stages of such evolution up to the Recombination Era, it was laws of quantum mechanics; during the Dark Ages it was gravitation; during the chemical evolution-diversification; and during the biological and human evolution—a process of distinctifying. The main event in the evolution of the Universe was the emergence of new levels of hierarchy, which together constitute the process of hierarchogenesis. This process contains 14 such events so far, and its dynamics is presented graphically by a very regular and smooth curve. The function that the curve presents is odd, i.e., symmetric about its central part, due to the similarity of patterns of the deceleration during the cosmic/chemical evolution (1st half of the general evolution) and the acceleration during the biological/human evolution (its 2nd half). The main driver of the hierarchogenesis as described by this odd function is counteraction and counterbalance of attraction and repulsion that take various forms at the different hierarchical levels. Direction and pace of the irreversible and inevitable increase of the Universe complexity in accordance with the general law of complification result from a consistent influence of all these factors.

## 1. Introduction

In my previous article, published in *Entropy* in 2015 with Prof. Alexander Levich [1] who untimely passed away and to whom I dedicate this article, we show that entropy, Shannon information, and algorithmic or Kolmogorov complexity are very proximate notions connected by a direct relationship with each other. Based on this similarity and on the concept of generalized entropy obtained by A.P. Levich on the basis of the theory of categories [2], we formulated the general law of complification. This law is a natural generalization of the 2nd law of thermodynamics that can be, strictly speaking, applied to thermodynamic systems only. According to it, the algorithmic complexity of the dynamical system described by categories with morphisms increases monotonically and irreversibly, tending to a maximum determined by external conditions. Respectively, this law, as well as the 2nd law of thermodynamics, determine the main direction of “the arrow of time” [3,4]. All the other physical laws of nature, which have structured the present world, are symmetrical relating to time and respectively describe the stationary world without any evolutionary elements. In addition, if the 2nd law of thermodynamics determines the direction of evolution in closed thermodynamic systems, then the general law of complification determines the direction of evolution of the whole Universe and all of its subsystems.

In this article, I attempt to reveal (i) how this general law of complification manifests itself at the different progressive evolution stages of our Universe; (ii) how it causes hierarchogenesis, as emergence of new levels of hierarchy; and (iii) the important although quite different roles of elements uniqueness in the first (pre-biological) and second (biological and human) phases of the Universe evolution. This hopefully will help to understand the peculiar mechanisms underpinning the general law of complification.

## 2. Life after Death (The “Thermal” One)

**Figure 1:**
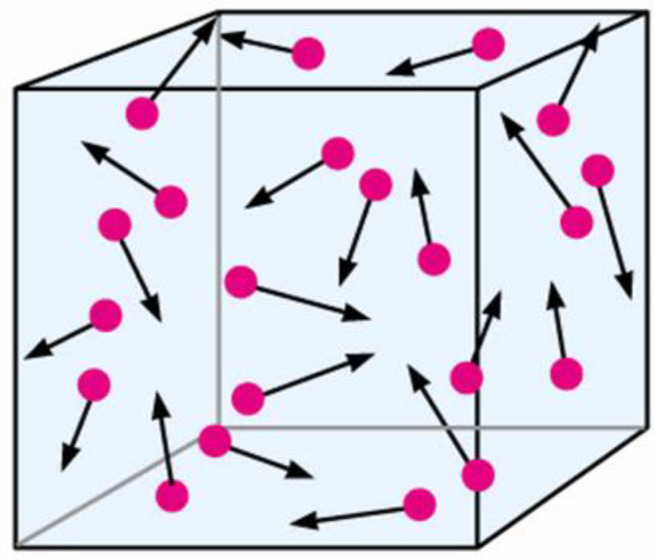
Point particles of the ideal gas in spatial projection distributed randomly and evenly on average. Directions of their velocity vectors are completely random while magnitudes of the vectors are randomly distributed around some average value proportional to the gas temperature.

Statistical entropy was originally determined by Ludwig Boltzmann [5] for an ideal gas, which consists of point particles that have mass and obey the laws of Newton’s mechanics. However, these particles present a limitation as regards interaction with one another in any way other than elastic collisions. Boltzmann considered such gas in bounded 6D space with the following axes: three spatial coordinates ***x***, ***y***, ***z*** and three coordinates of velocity: ***dx/dt***, ***dy/dt***, and ***dz/dt***. It is hard to perceive how gas molecules are distributed in this whole space, however, this segregated distribution could be done pretty easily for its 3D spatial and velocity projections. For states near thermodynamic equilibrium, spatial projection appears pretty trivial: Point particles distributed randomly and evenly, on average (Figure 1). When the gas states are far from equilibrium (e.g., all the particles are in the right half of the spatial projection), then their pretty special distribution will eventually and spontaneously become quite even and random. Correspondingly, after the removal of boundaries, the particles will fly apart further and further until their collisions stop.

**Figure 2:**
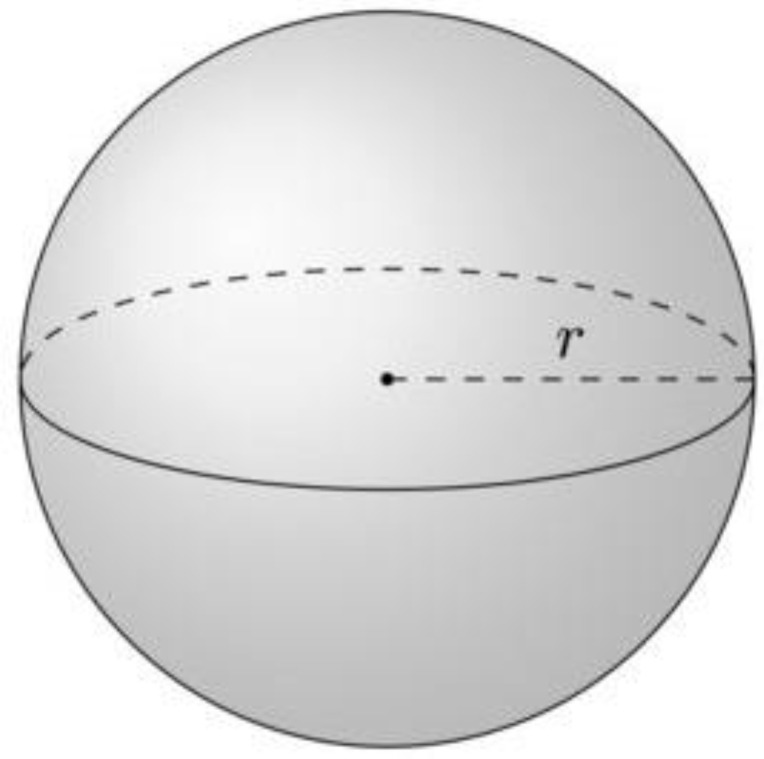
Point particles of the ideal gas in velocities projection distributed as a fuzzy sphere with average radius (***r***) proportional to gas temperature.

However, velocities projection is apparently more interesting. Gas near equilibrium is isothermal and presents on average the same magnitude of each point particle velocity vector (with the Maxwell distribution around the average value) while directions of the vectors are completely random. As a result, the distribution of point particles in this projection will be a regular fuzzy sphere with a center positioned at zero velocity (Figure 2). The sectioning of the sphere by its diameter will present the Maxwell distribution of particles with two maxima at the sphere surface.

With increasing temperature, the radius of the sphere will grow (and the density of particles in it will fall), and correspondingly, with its decrease, the radius will diminish (and the density respectively will grow) until the sphere reduces almost to a point near the absolute zero of temperature. In velocity projection, removal of the boundaries (in spatial projection) will not affect any alteration in the sphere. However, with the decrease of particle density in the spatial projection, and with ever rarer particle collisions, the particles constituting the sphere will demonstrate a decrease in their “jump” pattern from place to place until all of them will freeze at their positions at the last collision instant. This situation with the frozen particles can be considered as a kind of “thermal death”. Although we cannot visualize a complete shape and construct this model in 6D space, we could imagine a 3D projection with two velocity axes and one spatial axis.

**Figure 3:**
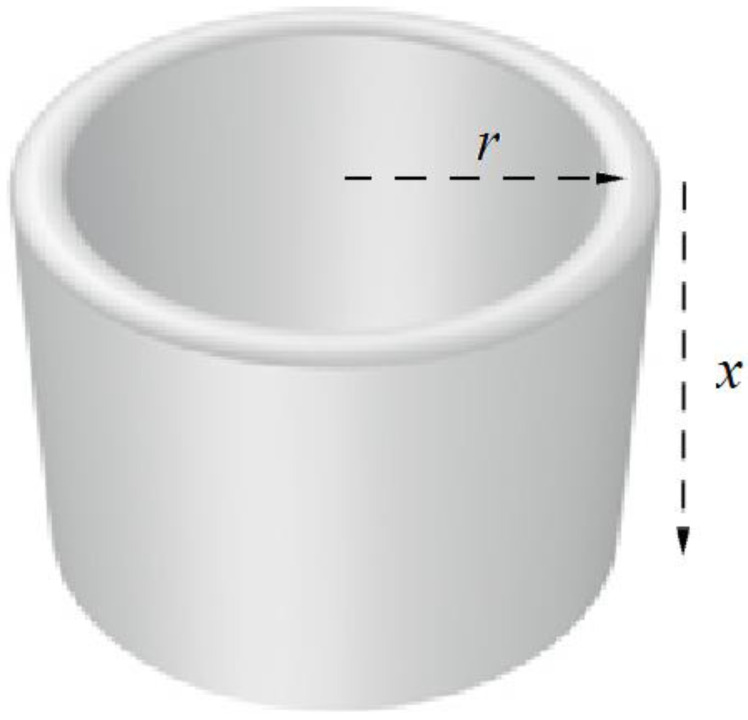
Projection of an image of ideal gas in a cube box in 6D spatial/velocities space into 3D space where two horizontal axes are velocities and vertical axis is one of spatial dimensions (where ***r***—average velocity of particles proportional to temperature and ***h***—length of the cube edge).

An image of Boltzmann’s model in a cubic spatial box with boundaries and not far from equilibrium is, in this projection, a fuzzy piece of tube (or more exactly, a hollow cylinder), where, radius (***r***) = the average velocity of particles and height (***x***) = the length of the edge of the box (Figure 3). This projection provides an adequate approximation of the 6D image, because the sphere in the 3D velocities projection is symmetrical, and the even and random distribution of particles in 3D spatial projection is the identical along each of the three spatial dimensions.

This model of an ideal gas in 6D spatial/velocity space allowed Boltzmann to infer the statistical determination of entropy:

***S*** = ***K_B_ lnW*** (1)

where, ***S*** is entropy, ***K_B_***-Boltzmann constant equals to 1.38 × 10^−23^ J/K^o^ and ***W*** is the thermodynamic probability that equals the number of microstates conforming to a given macrostate. Microstate in this determination refers to a specific distribution of particles in the 6D space with the precision of a very small product of ***Δx*** × ***Δy*** × ***Δz*** × ***Δ(dx/dt)*** × ***Δ(dy/dt)*** × ***Δ(dz/dt)***, which is chosen as a discrete cell of the space. Macrostate is a thermodynamic state, which is described by values of ***V*** (volume), ***P*** (pressure) and ***T*** (temperature). In addition, Boltzmann proved the H-theorem [5] that demonstrated how the laws of particle dynamics that are symmetrical in time leads to irreversible (in average) dynamics of H-function. In addition, this function can be interpreted as entropy assuming the equivalent probabilities of each microstate. Respectively, on average ***dS***/***dt*** ≥ 0 for an isolated system of ideal gas, and eventually it becomes 0 when entropy ***S*** reaches its extremum and all the particles of the system form a 6D hollow cylinder.

This model of an ideal gas can also be applied to provide an apt description of the real sufficiently rarefied (that allows to neglect interactions among the real atoms rather than collisions) monatomic gases, e.g., hydrogen. Our Universe, at the age of 380,000 years and also during the Dark Ages (175 million years until the ignition of the first stars) was a finite space occupied almost exclusively by rarified hydrogen (with a small admixture of helium). However, has the Universe been isolated?

The answer to this seemingly elusive question, in fact, seems quite obvious. Because Universe has no surrounding, it loses its property of any intersect to exchange energy or matter [6]. However, another perspective also exists. Arieh Ben-Naim [7,8] believes that we still do not know if the entire Universe, is, in fact, an isolated system. He might be right, and our novel ideas about the Universe may seem to our descendants naive, as were the ideas of William Thomson (Lord Kelvin) [9] about “heat death” of the Universe to us.

However, we, the humans, are deprived of definite knowledge about entities external to the Universe although the probability of such a phenomenon cannot be denied in full. However, for now, the thermodynamic isolation of the Universe is the most plausible hypothesis.

In consideration of the above, the Boltzmann’s model of an ideal gas is a relatively good approximation of our Universe during the Dark Ages. In addition, since the length of a 6D piece of the Universe tube demonstrated a continuous expansion, in this case, we should apply the variant that demonstrated removal of boundaries, with an exclusive correction of gradual decrease of the temperature (i.e., the average velocity of particles), according to the Big Bang theory [10]. According to this variant, the sphere slowly shrunk during the Dark Ages. A proximate consideration of the 2-velocities/1-space projection, as mentioned above, presents the Dark Ages Universe as a hollow cylinder with rapidly growing length (due to its spatial expansion) and gradually diminishing radius. The fate of such a piece of tube is pretty unenviable: Gas will increasingly rarefy, the atoms will stop colliding, the sphere of velocities will freeze, and the Universe as a whole will reach “the thermal (or heat) death”, as promised by William Thompson (Lord Kelvin) [9].

Fortunately, 13 billion years ago this phenomenon did not manifest itself, and the Universe continues to exist and evolve. Why? Gravitation reanimated our world at that time. Later such resuscitation was achieved by the diversification and distinctifying, discussed in the following sections. Now let us elaborate on the role of gravitation in the construct of the Universe’s fate.

The homogenous states with maximal entropy are stable only in the absence of gravitation. Sir Isaac Newton, as early as the 17th century, noted [11] that such states in gravitational systems may be unstable; this can happen due to the slightest perturbations of density. Such perturbations will continue to grow under the influence of gravitational forces. As a result, collisions among the particles will occur with increased frequency. The homogeneous state of matter, at early times, was evidently a state of low entropy and high free energy [12,13]. Later, the density fluctuations grew and grew, resulting in the formation of the first stars [14] and ignition of thermonuclear synthesis of helium from the hydrogen inside them under the enormous gravity induced pressure. This originated the local free energy sources in the Universe that, in fact, drastically redirected its evolution. The “cosmic dawn” came, the Dark Ages ended, and the “thermal death” was at least postponed.

## 3. From Identity through Diversity to Uniqueness

With the ignition of the first stars at the “cosmic dawn”, the situation in our Universe essentially changed. If during all the Dark Ages, it consisted almost exclusively of hydrogen (with a little bit of helium), thermonuclear fusion in the centers of stars yielded augmented helium as well as heavier chemical elements in the production. Such a Universe could not be approximated as an ideal gas with identical particles. I leave aside the stars, which in any case, consist of plasma, which is not a gas, and are extremely far from equilibrium. However, even interstellar matter cannot be regarded as a gas of identical particles because they ceased to be identical, owing to the star explosions that dispersed their matter with heavy elements into the environment.

**Figure 4:**
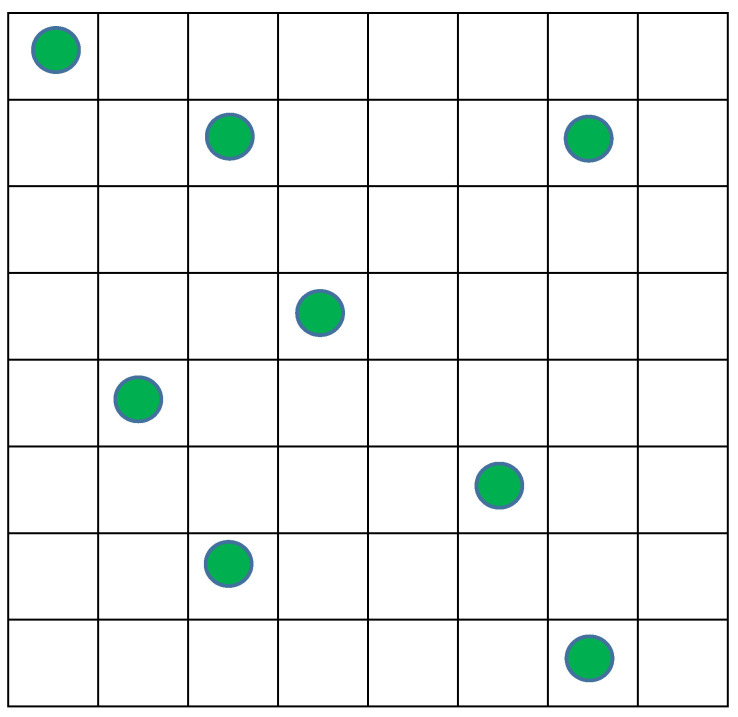
Random distribution of eight identical objects by 64 cells of the model space.

Let us begin from the simple model already used in our paper with Alexander Levich [1], to reach to an estimate of the real situation in the Universe about 13 billion years ago. The model is a very simple system that includes a discrete 2D space of 8 × 8 cells and ***N*** identical objects (***N*** = 8) distributed arbitrarily on these ***M*** cells and ***M*** = 64 (Figure 4).

For simplicity, we do not associate the objects on Figure 4 with any velocities and respectively exclude temperature ***T*** from the macrostate parameters. So, the macrostate is determined only by volume ***V*** that is 64 and pressure ***P*** that is 8/64 = 1/8. Hence, all the microstates with eight objects and 64 cells relate to the same macrostate, and their number equals to the number of combinations of eight elements by 64:

***W*_1_***= **M!/((M***− ***N)!*** × ***N!)*** = 64!/((64 − 8)! × 8!) = 4,426,165,368 (2)

**Figure 5:**
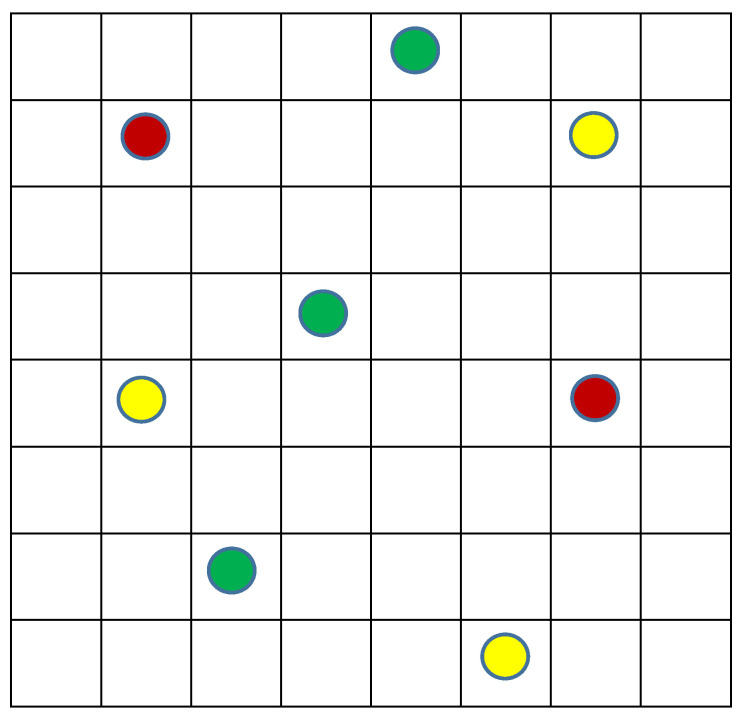
Random distribution of eight objects that belong to three types (red, yellow and green) by 64 cells of the model space.

Let us add now some diversity into our model and suppose that the eight objects belong to three different types rather than being identical (Figure 5):

In this case, the description of the macrostate includes additional parameters: number of types (***n***) and shares of each type (***N*_1_*, … N_n_***). These parameters for the macrostate on Figure 5 are: ***n*** = 3 and ***N*_1*(green)*_** = 3, ***N*_3*(yellow)*_** = 3 and ***N*_2*(red)*_** = 2. Respectively, any microstate on Figure 4 will correspond with several microstates on Figure 5 (with three types of particles) and the number of this microstates will be equal to perturbations with repetitions of all the objects divided to the product of such perturbations inside each of the types, i.e., ***N!/(N*_1_*!*** × ***N*_2_*!*** × ***N*_3_*! … N_n_!)***. Such a case related to Figure 5 gives: 8!/(3! × 2! × 2!) = 40,320/6 × 2 × 2 = 1680. Finally, the total number of microstates related to the macrostate displayed in Figure 5 will be:

***W*_2_***= **(M!/((M*** − ***N)!*** × ***N!))*** × ***(N!/(N*_1_*!*** × ***N*_2_*!*** × ***N*_3_*! … N_n_!))*** = ***M!/((M*** − ***N)!*** × ***N*_1_*!*** × ***N*_2_*!*** × ***N*_3_*! … N_n_!)*** = 64!/(56! × 3! × 2! × 2!) = 7,435,957,818,240 (3)

This number ***W*_2_** is 1680 times more than ***W*_1_** for the case of identical particles (Figure 4) even for our very tiny model system. With increasing ***M***, ***N*** and ***n***, the relation of the number of the microstates with particles belonging to different types to such a number with identical particles (***W*_2_/*W*_1_**) grows and at an extremely rapid rate.

**Figure 6:**
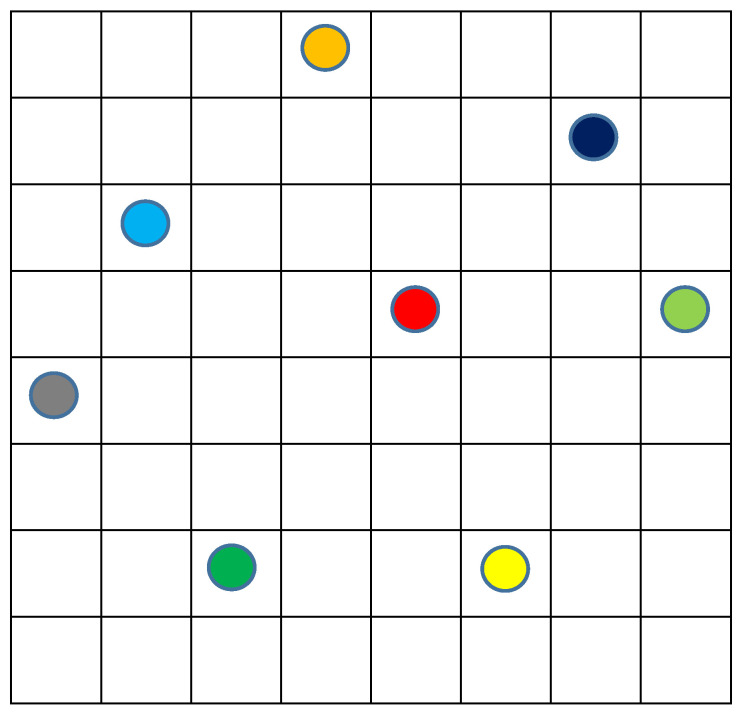
Random distribution of eight unique objects that belong to eight types (colors) by 64 cells of the model space.

Consider now uniqueness as the extreme option of diversity. In this case, as presented in Figure 6, number of types (***n***) equals to the number of particles (***N***). In other words, the system does not have even two identical particles. The number of microstates for this option equals (for each spatial distribution from ***W*_1_**) to perturbations without repetitions of all the objects or simply ***N!***. Respectively, the total number of microstates related to any given position including ones displayed in Figure 6 will be 8! = 40,320. As for the total number of microstates related to the macrostate presented in Figure 6, it will be:

***W*_3_** =***(M!/((M****− **N)!*** × ***N!)))*** × ***N!****= **M!/(M** − **N)!*** = 64!/56! = 178,462,987,637,760 (4)

Using Boltzmann formula for calculation entropy of this simple model, we will get the following values (in units of the Boltzmann constant):

***S*_1_***= **lnW*****_1_** ≈ 22.21; ***S*_2_***= **lnW*****_2_** ≈ 29.64; and ***S*_3_***= **lnW*****_3_** ≈ 32.82 (5)

Because ***W*_3_*/W*_1_** *= **N!***, the difference between ***S*_3_** and ***S*_1_** is ***lnN!***, or, applying Stirling approximation, ***N*** × ***ln N****** − N***.

On the other hand, if we calculate Kolmogorov complexity [15] ***K(s)*** for the macrostate displayed on Figure 4, we will get (in bits):

***K(x)****= **N*** × ***log*_2_*M*** = 8 × ***log*_2_** 64 = 48 (6)

The same calculation for Figure 6 gives:

***K(x)****= **N*** × ***log*_2_*M****+ **N*** × ***log*_2_*N*** = 48 + 24 =72 (7)

In both cases, it shows about a 1.5 times increase from identical to unique particles; both of these second terms demonstrate a very expedited increase with corresponding increasing ***N***; and they practically equal one another for big ***N***.

Applying this simple model to the real systems, we should, foremost, determine for them the notions of (i) identity, and (ii) uniqueness essentially used by the model. Usually, the species of identical physical particles include, but are not limited to elementary particles, atomic nuclei as well as atoms and molecules. However, if the quantum mechanical identity is complete and obvious, the identity of such classical objects as large enough molecules of the same substance can, in fact, be questionable. For instance, with the inclusion of extrinsic parameters (such as position or velocity) in addition to intrinsic (like mass or composition) into particle properties, almost all the atoms or molecules will be considered as unique. In the context of this article, such an approach is seemingly unproductive, because it deprives atoms and molecules of their self-identity and any atom that changes its position or velocity becomes another entity. However, refraining from exploring these general problems, I will consider identity in a quite limited and clear sense:

Two or more physical systems can be considered identical if they consist of exactly the same set of elements or subsystems and these elements form exactly the same structure.

In other words, for the positioning of a physical system in correspondence to a graph having nodes that relate to the system elements while its edges relate to its structure as links between the elements, the systems must be considered identical if and only if such graphs are isomorphic. Considering the structure in both these definitions allows us to consider isomers, including enantiomers, as not identical. As well, the same isomers of the same molecules are not identical if even one pair of their atoms at the same position is represented by different isotopes.

Let us return now to the chemical evolution of the Universe, which developed first in the interstellar dust, and subsequently in the planetesimals and finally on the planets. Nuclei of heavy elements, synthesized by stars of the Population I, produced several dozens of chemical elements. These, in turn, reacted with each other, forming hundreds of the simplest, then thousands of more complex chemical compounds and eventually millions of heteropolymers.

Each such step led to a jump of diversity (***D*** *= **n/N***), that was equal to 0 for hydrogen gas and became 1 for unique living droplets (that we consider in the following section) because the probability of finding two of them with exactly the same set of polypeptides or nucleic acids is negligible. However, between these jumps, diversity also monotonically increased as the molecular structure becomes more complex. The second term of Kolmogorov complexity (***K***) (see formula 7) determined by diversity and only by it, grew from 0 to enormously huge values proportional to ***N*** × ***logN*** where ***N*** is several orders of magnitude greater than the Avogadro number (6.022 × 10^23^). This provided excess scope for increasing complexity, in comparison with sets of identical objects with the same cardinality. Thus, during this stage of the general evolution of the Universe, diversification as growth of diversity was the main driver of complification.

## 4. Growth of the Uniqueness Degree: The Biological Evolution and Beyond

Apparently, the first unique objects with diversity equal 1 were living droplets. In fact, the first living systems appeared, by estimates of Alexei Sharov and Richard Gordon, based on backward interpolation of the exponential increase of the genome complexity over its history in accordance with Moore’s Law [16,17], 9.7 ± 0.3 Ga (Giga-or billion years ago), i.e., long before the emergence of our Earth. We can very vaguely imagine the structure of these droplets. All the details and discussion on their possible structures one can find in the original research [16] and especially [17]. However, definitely none of the droplets were completely unique in their sets of macromolecules (heteropolymers). In other words, its diversity (***D*** *= **n/N***) is 1, i.e., ***n*** (number of types inside which all the individuals are identical) equals to ***N*** (number of individuals) because each such type, in the absence of identical droplets, contains only one individual.

Did the complification stop after this? No, it did not.

**Figure 7:**
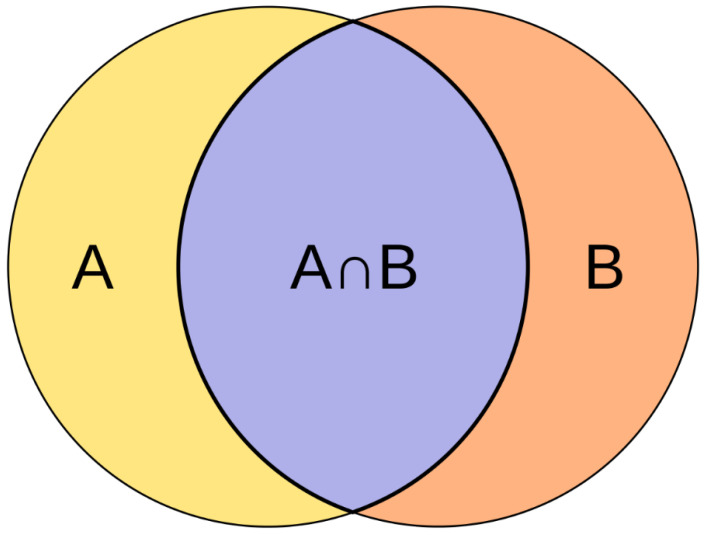
Jaccard distance as a measure of how dissimilar two sets are. For the above example, the Jaccard distance is (|***A*** ∪ ***B***| − |***A*** ∩ ***B***|)/|***A*** ∪ ***B***| (from en.wikipedia.org, https://en.wikipedia.org/wiki/Jaccard_index).

Essentially, uniqueness means the absence of identity of objects but does not imply their complete difference. For instance, two prokaryotic cells even though very similar, are unique. This similarity can be measured based on Kolmogorov complexity mentioned above [15], ***K(s)*** *= **Min(d(s))*** ∈ ***{d(s)}*** where ***d(s)*** is a length of complete binary description of system ***s***. The longer the coinciding part of the minimal but complete descriptions of two objects, the greater similarity of these two unique objects. A measure of uniqueness, on the other hand, can be determined as the length of the non-coinciding part in these descriptions that is actually Jaccard distance that equals to the difference of the sizes of the union and the intersection of two sets divided by the size of the union (Figure 7). So, the uniqueness of two unique objects ***i*** and ***j*** is:

(8) $$U(i,j)=(K(s_{i})+(K(s_{j}))-Min(d(s_{i}\cap s_{j})))/(K(s_{i})+K(s_{j}))$$

Respectively, distinctifying, that I determine as increasing (or decreasing, if it is negative) of the uniqueness, is ***dU/dt***.

Finally, in a general case, the distinctifying equals to,

(9) $$\frac{dU}{dt}=\left(\frac{\sum_{i=1}^{n}\sum_{j=1}^{n}K\left(s_{i}\right)+K\left(s_{j}\right)-Min(d\left(s_{i}\cap s_{j}\right)}{2\times \sum_{n}K(s_{i})}\right)/dt$$

where ***i*** ≠ ***j*** and ***n*** is a number of unique objects.

Such distinctifying ***(dU/dt)*** began to play the imperative role of the main propellant of complification in a process of biological evolution subsequent to the emergence of the first biological entity and the first completely unique systems. The uniqueness ***(U)*** of two and more objects is calculated by the formula presented above. It changes from 0 (for identical objects) to 1 (when descriptions of the objects have absolutely nothing in common). On the first glance, a range of uniqueness includes the range of diversity: when diversity reaches 1, uniqueness continues to grow. However, actually, diversity and uniqueness are two separate dimensions of complexity. Corresponding to the growth of diversity, the number of unique types of identical objects also increases while the growth of uniqueness implies increasing of dissimilarity of the unique types or objects. Furthermore, when the number of unique types becomes equal to the number of objects (i.e., diversity reaches a maximum), uniqueness continues to grow due to the increasing of the uniqueness degree.

The simplest living objects known currently are the prokaryotic cells. Their interspecies similarity despite their individual uniqueness is quite evident while their structure is very simple: ribosomes and genetic material (DNA/RNA molecules) in cytoplasm surrounded by plasma membrane. The eukaryotic cells that appeared about two billion years later have a more complex structure and are less similar. Their structure includes (in addition to prokaryotic elements) nucleus, nucleoulis, multiple chromosomes, mitochondrias, endoplasmic reticulum, Golgi appratus, lysosomes, vacuoles and chloroplats (in plant cells). However, in addition to this spatial structure, eukaryotic cells present a pronounced temporal structure in the form of a mitotic cycle that consists of prophase, metaphase, anaphase, telophase and interphases G^1^, S, and G^2^. Resultantly, the eukaryote uniqueness was and is drastically higher than that of prokaryotes. Subsequently, after more than a billion years, multicellular organisms as systems of eukaryotic cells appeared. This led to an enormous variety of spatial structures in combination with a far more complicated temporal structure that manifested itself in the embryogenesis [18] or life cycle (in the case of metamorphosis). Thus, uniqueness, as well as complexity, acutely soared.

Such an increase in complexity in a row prokaryotes > eukaryotes > multicellular organisms was shown and discussed by Maynard Smith and Eörs Szathmáry [19]. However, although the variety of species and higher taxa is very wide; the intraspecies variability has remained very low, and therefore, biologists must necessarily tag individuals of the same species (even mammals and birds) in order to distinguish between them. However, after the appearance of agroecosystems, i.e., artificial ecosystems, not only have the variety of communities in the same climatic zone essentially increased but, due to artificial selection, there has been an unprecedented increase in the intraspecies variability. It is enough to recall the different breeds of dogs, cats or chickens. At the same time, the temporal structure of agroecosystems gained an additional level: Ancient farmers added a crop rotation to natural circadian and seasonal dynamics. However, most importantly, this step was accompanied by the appearance of reasoning and creative humans, who, in fact, initiated the next, human stage of the general evolution.

Soon afterward, in about 9000 years, farmers with their quite different agroecosystems (fields, herds on pastures, groves, gardens, kaleyards, apiaries, and so on) came together and united into nations and countries. Each of these nations elaborated their own indigenous customs, religions, and cultures, which, subsequently led to the large variety of the human being. This variety, however, is very insignificant compared to the variety of our minds. We reflect the world around us in our own subjective mirrors and the curvature of each has an individual uniqueness. Yet this uniqueness is still much less than 1 and this opens the scope for future evolution.

## 5. Staircase of Hierarchogenesis: From “Quark Soup” to Globalization 

The evolution of our Universe has never followed a smooth and consistent pathway. It has been proliferated with inflection points, the emergence of new functionalities, catastrophes, and so on. Events of so-called hierarchogenesis [20,21] were the rarest and the most important of these escalations. Such events are characterized by the appearance of a new level of hierarchy. The notion of hierarchy has several different meanings, starting from the original, churchly version, according which people or groups are ranked one above the other based on status or authority [22]; that is obviously not our case. This presents the impending need to explicitly define what we mean by a hierarchogenetic event in the context of the evolution of the Universe.

An event can be considered as hierarchogenetic if it results in the appearance of a system that:

1. Can exist by itself, not only as a part of a super-system on upper hierarchical level(s);
2. Consists of subsystems belonging to one or more lower hierarchical levels;
3. Its subsystems are of several types that radically differ from one another;
4. Interrelations between these subsystems lead to the emergence of an entity that did not exist before, i.e., a novelty.

The first of the conditions above excludes systems such as free radicals, cell organelles, or organs (and systems of organs) of multicellular organisms. The second condition excludes hierarchical systems in their original, churchly sense. For example, alpha male in a flock of monkeys is the highest level of hierarchy but it does not consist of beta males, females, juveniles, etc. The third condition excludes systems that consist of the monotypic or almost monotypic subsystems like homopolymers, colonies, populations (in ecological sense), or some multicellular prokaryotes [18,23]. Furthermore, the fourth condition does not allow for the consideration, for instance, of each of the multiple emergences of multicellularity in different clades [23,24,25] as separate hierarchogenetic events, as opposed to eukaryotes that appeared probably only once in the history of life [26,27]. Thus, in adherence to this condition, the appearance of eukaryotes and multicellular organisms should be considered as only one hierarchogenetic event in each case.

Correspondingly, the application of our definition to the whole history of the Universe reveals only 15 hierarchogenetic events with two branches: (i) Cosmic and (ii) Substance. These events are listed in Table 1 with the time of emergence, duration, and areas of science related to them. 

Particularly, the numbers in the 3rd column of Table 1 are approximate or average for interval values found in the different sources and, based on these numbers, the 4th column ones have been calculated. Time of the Big Bang (as a zero point) was assumed equal to 13.8 ± 0.02 Ga, i.e., billion years ago [28]. The appearance of quark-gluon plasma (“quark soup”) and hadrons were estimated as 10^−12^ and 10^−6^ s after the Big Bang, respectively [29]. First nuclei appeared from 1 s until after a few minutes of the Universe’s existence [30]. So, time from the Big Bang to each of these first three steps is equal practically to zero (in our gigayears time scale).

In continuation, the appearance of the first atoms in the Recombination Era is dated 380 ± 50 thousands of years [31] after the Big Bang. First stars appeared in 13.78 Ga, or more exactly −175 ± 75 My (million years) after the zero point [32]. However, the latest observations [14] showed the existence of the first stars 180 My after the Big Bang that essentially narrowed the previously computed estimations. This time stamp has been used in Table 1. The time interval of the appearance of the first galaxies was pretty wide: 150 My − 1 Gy after the beginning of the Universe [33]. Furthermore, I originally chose for this step the middle value −0.575 ± 0.425 Gy. However, in January of 2018 NASA published report [34], which mentioned the finding of one of the Universe’s oldest galaxies, which formed only 500 million years after the Big Bang. So, I chose 0.49 ± 0.01 Gy as the date of galaxies appearance.

This appearance of galaxies concluded the cosmic branch of the general hierarchogenesis because clusters and superclusters of galaxies that formed about 3 and 5 Gy (billion years) after the Big Bang, respectively, are not in compliance with our 3rd requirement for hierarchogenetic event. In fact, these clusters and superclusters are just a kind of “colonies” of galaxies rather than real novelties. The following hierarchogenetic steps belong to the second (Substance) branch moved away from the main one about 1–2 Gy of the Universe’s age. Furthermore, in particular, this step started with the appearance of substance as interstellar dust in a form of heteroatomic molecules. During the study, to identify a value of the time to pinpoint the first relatively complex (heteroatomic) molecules emerged, was the most complicated task. I estimated it based on the time of the interstellar dust appearance, i.e., as 1.1 ± 0.3 Gy after the Big Bang [34].

Unfortunately, no data at all is available to signify the time of heteropolymers appearance. As for the appearance of the first living droplets (the first extraterrestrial living systems), this happened, as we mentioned above, at 9.7 ± 0.3 Ga or 4.1 ± 0.3 Gy after the Big Bang [16,17]. Prof. Gordon and myself [35] analyzed numerous data on the time when the first prokaryotic and the first eukaryotic cells had emerged. Finally, we got the following estimates for these hierarchogenetic events: 4.05 ± 0.25 Ga and 2.1 ± 0.6 Ga or 9.75 ± 0.25 and 11.7 ± 0.6 Gy of the Universe history, respectively.

The next step was to identify the appearance of eukaryotic multicellular organisms. This happened 765 ± 25 million years ago [36] or 13.035 ± 0.025 Gy after the Big Bang. The two following steps (appearance of agroecosystems and states/nations) happened 14,000 ± 4000 [37] and 5100 ± 100 [38] years ago, respectively. However, these points, similar to the very first events, are practically indistinguishable (in our gigayears time scale) from the present in this case.

Table 1 includes two branches of the main hierarchogenesis: The primary branch (Cosmic) followed from the Big Bang, up to the formation of galaxies. The second branch (Substance) started with the appearance of substance as interstellar dust in a form of heteroatomic molecules. These molecules gradually gained increasingly complex structures (presumably on the surface of the planets), combined into heteropolymers (macromolecules) and then subsequently originated the following hierarchical levels of living droplets, prokaryotes, eukaryotes, etc. Correspondingly, the time of occurrence of each of these steps was identified based on the best approximate estimation from the sources mentioned above. The only exception to this is the time of appearance of the macromolecules, which cannot be estimated, even approximately. Stanley Salthe [39,40] demonstrates a similar approach to understanding the hierarchy. He calls it the compositional hierarchy and opposes it to classification hierarchy, widely used in biology for hundreds of years, but does not reveal its branches.

Of course, in the addition to the main, substance branch:

**Monomers** ⇨ **Heteropolymers/Macromolecules** ⇨ **Living Droplets** ⇨ **Prokaryotic Cells** ⇨ **Eukaryotic unicellular organisms** ⇨ **Multicellular organisms** ⇨ **Agroecosystems** ⇨ **Nations/States** ⇨ **Noosphere (?)**

the other branches are also evident, like:

**Interstellar dust** ⇨ **Planetesimals** ⇨ **Planets** or 

**Living organisms** ⇨ **Ecosystems** ⇨ **Biosphere**

where, in fact, developing in parallel with the principal direction of hierarchogenesis. However, they do not belong to the mainstream of the Universe evolution, at least from the human perspective.

The 15th step in Table 1 is noosphere, i.e., the sphere of human thoughts, as a possible candidate for the next hierarchogenetic event. The concept of the noosphere was formulated by Teilhard de Chardin in 1922 [41] and then developed by himself in his main treatise, “The Phenomenon of Man” [42] and by Vladimir Vernadsky [43] as the possible next stage of human evolution. However, the particular form of noosphere in which it will be realized as the next hierarchogenetic step is hard to predict, because the evolution at and after a bifurcation point cannot be predicted just before it, where we obviously are now. Yet we already can observe some essential signs of the noosphere appearance: Globalization, the Internet, Social Media, Crowd Thinking, etc.

**Figure 8:**
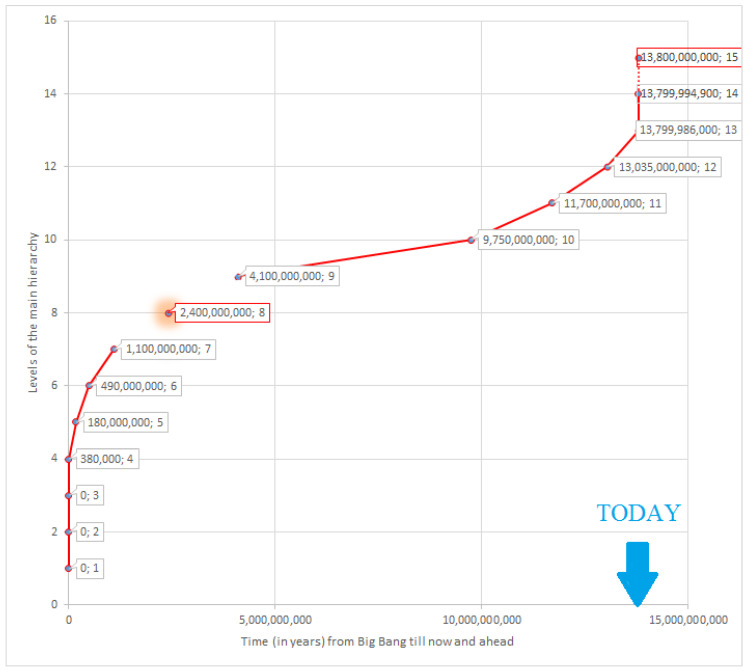
Dynamic of successive steps of the main hierarchogenesis of the Universe (see Table 1) since the Big Bang. X axis is a timescale of the Universe evolution in gigayears while Y axis is a sequential series of hierarchial event. Scale of axis Y is arbitrary, but it is substantially that distances between any two adjacent events are the same. Points: 1—quarks, 2—hadrons, 3—nuclei, 4—atoms, 5—stars, 6—galaxies, 7—heteroatomic molecules, 8—heteropolymers/macromolecules, 9—living droplets, 10—prokatyotic cells, 11—unicellular eukaryotic organisms, 12—multicellular organisms, 13—agroecosystems, 14—nations/states, 15—noosphere. Step 8 (macromolecules) cannot be confidently dated and the number related to this point is an interpolation. Point 15 (noosphere?) relates to the future, and the dotted line between points 14 and 15 describes probable prediction.

Placing positions of the timestamps related to all the hierarchogenetic steps (the 3rd column in Table 1) onto the timeline from the Big Bang until today (and a little bit in the future), we obtain a very regular and smooth curve (Figure 8). This curve can be described as odd, i.e., symmetric about its central part function. This symmetry follows from the similarity of patterns of the deceleration during the cosmic/chemical evolution (1st half of the general evolution) and the accelerating during the biological/human evolution (its 2nd half). In one of my previous articles [44], I supposed a simple mathematical model based on a semantic approach that optimally approximates the curve in Figure 8. However, such considerations are out of the scope of the current article.

As mentioned above, I am limited in identifying when the first heteropolymers or macromolecules (e.g., proteins or nucleic acids) emerged although this definitely happened after the formation of monomers (12.7 ± 0.3 Ga) [34] and before the emergence of the first living droplets that took place, accordingly (9.7 ± 2.5 Ga) [17]. As a result, the line on Figure 8, 12 shows a gap between 1.1 and 4.1 Gy since the Big Bang. However, all the other points almost ideally lie on our smooth curve, and this allows to assume the most probable position of the missed point and respectively to estimate appearance of macromolecules about 11.4 ± 0.1 Ga or 2.4 ± 0.1 Gy after the beginning of the Universe, keeping in mind that this is only an approximate value. On the other hand, I could try to extrapolate the next hierarchical step (noosphere or, perhaps, something else). Unfortunately, such extrapolation can be done only very roughly due to extremely high speed of the hierarchogenesis in its very beginning and approaching today. Taking into account this symmetry, we can expect the onset of the 15th hierarchogenetic step in about a few hundred years, if not decades.

Of course, this list of 15 hierarchogenetic event is not final and could be slightly modified [20,21,45]. Jagers op Akkerhuis [20,21] did not include levels of nuclei, macromolecules, living droplets, agroecosystes and nations/states while Tyler Volk [45] did not include macromolecules and living droplets, either, but added animal social groups and tribal metagroups that I have not considered because they do not match the 3rd condition of hierarchogenetic event. In addition, both authors ignore the last two steps of the main, cosmic direction of the Universe evolution, i.e., stars and galaxies. Generally, however, Table 1 with the 15 rows give us an approximation to the number of hierarchogenetic events and illustrates a general picture of the hierarchogenesis as the main staircase of material evolution. Each such event accompanied by a sharp increase of complexity resulting from an emergence on the new hierarchy level evidenced a set of interactions between systems of the previous level. Respectively, the complexity of a system on the new hierarchical level is a sum of the complexities of its subsystems and the complexities of links between these subsystems:

(10) $$K\left(S\right)=\overset{n}{\underset{i=1}{\sum }}K\left(s_{i}\right)+\overset{n}{\underset{i=1}{\sum }}\overset{n}{\underset{j=1}{\sum }}K\left(L_{ij}\right)$$

where ***K(S)***—the complexity of system ***S*** on the new upper level of the hierarchy, ***K(s_i_)***—the complexity of ***i***-th subsystem on its previous lower level, and ***K(L_ij_)***—the complexity of a link between ***i***-th *and* ***j***-th subsystems **s** on the previous level.

Because the number of possible links, that equals ***n*(*n* − 1)/2**, is much larger than the number of subsystems ***n***, the complexity due to hierarchogenetic step showed a multifold increase. Surely, the complexity grew between the hierarchical steps, too, due to quantum mechanical laws, gravity, diversification, and distinctifying, but this growth was far slower and smoother. In addition, it has been the hierarchogenesis that determines the principal pace and direction of matter evolution. Furthermore, each step in this hierarchogenesis is essentially a consolidation of a set of essentially different systems into a system of a higher level of the hierarchy. However, this raises a question: What could be the basis of such consolidation?

The answer to this question, although probably too general, is quite obvious: Any consolidation cannot be achieved without a kind of attraction among the consolidated systems. I will attempt in this paper to concretize this answer and discuss the meaning of attraction concepts at various levels in the next section.

## 6. Complification Driven by Counteraction and Counterbalance of Attraction and Repulsion 

As supposed above, the general hierarchogenesis has been evidenced in some or the other construct of attraction among the systems of the previous hierarchogenetic level. Without such attraction, these systems could not obviously originate a super-system belonging to next, new level of the main hierarchy. However, the physical, chemical, biological, anthropological, political or economic implementations of this attraction on the 15 different levels of the main hierarchy are quite diverse.

For quarks in hadrons and hadrons in nuclei, it is strong forces; for nuclei and electrons in atoms-electromagnetic forces; for ionized atoms in stars and stars in galaxies-gravity; for atoms in molecules (monomers) and monomers in heteropolymers-ionic, covalent, and hydrogen chemical bonds.

Attraction in the biological systems takes the form of symbiosis. The very term “symbiosis” was proposed almost 150 years ago by Heinrich Anton de Bary [46,47]. Then, after a quarter of a century, Peter Kropotkin [48] came to the conclusion that the symbiosis in the form of “mutual aid” plays an essential role in the evolution of multicellular organisms. In addition, Konstantin Merezhkovsky [49] more than a hundred years ago formulated the theory of symbiogenesis, which claims that eukaryotes appeared as a result of the symbiosis of different prokaryotic cells. However, more than 40 years passed before these ideas became well-known, which was subsequent to the publishing of Lynn Margulis’s famous book Origin of Eukaryotic Cells that gave the endosymbiotic theory a new life [50]. Only in the 1970s, this theory gained at last its wide recognition, and symbiosis as a biological kind of attraction, was recognized as one of the main factors of biological evolution [51]. This allows us to consider symbiosis as the leading driver of biological hierarchogenesis.

For agroecosystems, this attraction, as opposed to all the other cases, manifested itself in an asymmetric pattern. It took the form of selection of plant and animal species that could function and serve as a reliable and replenished source of food, by ancient humans, in the ecosystems where they not only lived but also reconstructed them for their needs. However, none of these species demonstrate, at least at the beginning, any attraction to the humans. As regards the nations and states—it was an attraction between these agroecosystems, or more exactly farmers, i.e., families and Neolithic settled tribes, in the face of external threats in the form of raids by nomad tribes that have not yet moved to a settled way of life. Furthermore, with this understanding and aligned evolution, eventually, noosphere can be understood as a successful completion of globalization. This event will be able to emerge only as a result of an attraction between the states and nations for solving global problems that threaten the very existence of the humanity. Hopefully, this next hierarchogenetic step will happen before it is too late.

Concurrently, each of these types of attraction is aligned with some sort of repulsion that does not allow the systems to merge into one instead of forming a new level of hierarchy. The primary and the most important repulsion was the Big Bang that commenced the enlargement of the Universe itself and projected attraction (that has been acted in the opposite direction) as the main driver of the matter evolution. The other, more particular kinds of repulsions also have been understood to essay diverse nature/behavior for different hierarchical levels.

For hadrons in nuclei, it is the repulsion of the electrical charges. For the stars, it is the energy of thermonuclear synthesis that prevents the stars from collapsing into a black hole until thermonuclear fuel, first of all, hydrogen, is not exhausted. For galaxies, it is centrifugal force of their rotation that prevents the galaxy stars from immediate falling into the black hole that is usually located in the centers of galaxies. For atoms in monomers and monomers in heteropolymers, it is the Pauli Exclusion Principle that prevents two or more identical fermions, including electrons, from occupying the same quantum state within any quantum system including molecules. As a result, atomic nuclei in each molecule are separated by electronic clouds populated by not more than two electrons with opposite spins at each energy level. This, on one hand, compensates the electric repulsion between the positively charged nuclei and, on the other hand, does not allow them to get essentially closer than a sum of atomic electron clouds radii.

For prokaryotic, unicellular eukaryotic, and multicellular biological organisms, the structures rather than powers play the same role. In prokaryotic cells, plasma membrane together with ribosomes, cytoplasma, and genetic material are the elements that determine their external and internal structure. In addition, just as electronic clouds do not allow the atomic nucleus and electrons to merge into one, these structures form interactions between the elements preventing a complete fusion of primitive living drops. In eukaryotic cells, it is endoplasmic reticulum and intercellular membranes that keep cell organelles at a distance and provide their proper interactions. Finally, exterior cell membranes, connective tissue, ligaments and even elements of skeleton act in a direction opposite to symbiotic attraction among the cells in multicellular organisms.

In addition, a kind of repulsion exists among the organisms. It is the law of competitive exclusion formulated by Georgy Gause [52] according to which two species competing for the same limiting resource cannot coexist or, alternatively, they cannot occupy the same ecological niche. This in fact places Gause’s principle in direct contradiction with (and yet makes it complementary to) the law of congruous attraction [53] that I formulated more than 30 years ago.

For agroecosystems, it is the resistance of animal species to domestication and instability of one-crop agricultural systems. Respectively, the ancient humans had to graze cattle to build fences and to clean the fields of weeds, i.e., “by the sweat of their faces they ate bread”. For states and nations, there were inevitable contradictions, always arising between neighbors, be they families, tribes or countries. Finally, for the noosphere, we can witness with our own eyes all the political, economic, cultural, and religious obstacles hindering the integration of all the humanity into the one super-system that probably constitutes the next step of the hierarchogenesis. Obvious signs of such a supersystem emerging can be seen in such organizations as The Global Brain [54] or Global Mind Share [55].

This permanent counteraction and balance of attraction and repulsion have, in fact, not allowed the process to stop by achieving some kind of complete integration. On the contrary, the integration at each step of the hierachogenesis was not perfect, due to repulsion, and this has always opened possibilities for further evolution.

## 7. Conclusions

- During the Dark Ages, the Universe could be approximated as “ideal gas” and had the 6D tubular shape in a space of coordinates and velocities of hydrogen atoms.
- Gravitation did not allow this gas to reach maximal entropy and complexity and ignited the stars that produced heavy elements and started chemical evolution.
- Chemical evolution, as well as the previous cosmic evolution, developed from identity to uniqueness and consistently decelerated until the appearance of the first living systems that were the first completely unique objects.
- Chemical evolution was driven by a process of diversification that allowed complexity to rise far beyond maximal values attainable for a gas of identical particles.
- Consistent distinctifying of the unique objects has been accompanied by practically unlimited and accelerated growth of complexity.
- A function that describes the whole evolution of the Universe is odd, i.e., symmetric about its central part, due to the similarity of deceleration pattern during the cosmic/chemical evolution (1st half of the general evolution) and the acceleration one during the biological/human evolution (its 2nd half).
- The main event in the evolution of the Universe was the emergence of new levels of hierarchy-hierarchogenetic steps.
- There were 14 such steps of hierarchogenesis so far, from “quark soup” until the present process of globalization, i.e., the process that is leading to the next, 15th step in the very near future.
- This hierarchogenesis is driven by counteraction and counterbalance of attraction and repulsion that adopt various forms at different hierarchical levels.
- All these processes lead to an irreversible and inevitable increase of the Universe complexity in accordance with the general law of complification.

## Figures and Tables

**Table 1:** Hierarchogenetic branches and steps in material evolution of the Universe (question marks denote interpolated (macromolecules) and extrapolated (noosphere) values.

Hierarchogenetic Branch	Hierarchogenetic Step	Time after Big Bang (In Years)	Duration (In Years)	Related Area(s) of Science
Cosmic	1-Appearance of the rest mass and light particles (quark-gluon plasma)	3.17 × 10^−20^	3.17 × 10^−20^	Elementary particle physics
	2-Appearance of hadrons (heavy particles)	3.17 × 10^−14^	3.17 × 10^−14^	Physics of strong forces
	3-Appearance of nuclei	3.17 × 10^−7^	3.17 × 10^−7^	Nuclear physics
	4-Appearance of atoms	3.80 × 10^5^	3.80 × 10^5^	Quantum mechanics, Spectrometry
	5-Appearance of stars	1.80 × 10^8^	1.80 × 10^8^	Astrophysics
	6-Appearance of galaxies	4.90 × 10^8^	3.10 × 10^8^	Astrophysics
Substance	7-Appearance of heteroatomic molecules (monomers)	1.10 × 10^9^	6.10 × 10^8^	Chemistry
	8-Appearance of macromolecules (heteropolymers)	2.40 × 10^9^(?)	1.30 × 10^9^(?)	Biochemistry
	9-Appearance of living droplets	4.10 × 10^9^	1.70 × 10^9^(?)	Biochemistry of RNA/protein/coenzyme worlds
	10-Appearance of prokaryotic cells	9.75 × 10^9^	5.65 × 10^9^	Microbiology
	11-Appearance of eukaryotic cells with mitotic cycles	1.17 × 10^10^	1.95 × 10^9^	Protistology
	12-Appearance of eukaryotic multicellular organisms with continuing differentiation [23], and thus embryogenesis	1.30 × 10^10^	1.34 × 10^9^	Embryology
	13-Appearance of artificial environment (agroecosystems), i.e., Neolithic revolution	1.38 × 10^10^	7.65 × 10^8^	Anthropology, Agronomy, Veterinary
	14-Appearance of nations and states with armies and governments	1.38 × 10^10^	8.90 × 10^3^	History, Economics, Politics
	15-Appearance of noosphere	1.38 × 10^10^	5.10 × 10^3^(?)	Crowd Thinking, Social Networking, Politics
